# CK2 Is the Regulator of SIRT1 Substrate-Binding Affinity, Deacetylase Activity and Cellular Response to DNA-Damage

**DOI:** 10.1371/journal.pone.0006611

**Published:** 2009-08-14

**Authors:** Hyeog Kang, Jae-Won Jung, Myung K. Kim, Jay H. Chung

**Affiliations:** Laboratory of Biochemical Genetics, National Heart Lung and Blood Institute, National Institutes of Health, Bethesda, Maryland, United States of America; University of Minnesota, United States of America

## Abstract

SIRT1, an NAD^+^ (nicotinamide adenine dinucleotide)-dependent deacetylase, protects cells from stress-induced apoptosis, and its orthologues delay aging in lower eukaryotes. SIRT1 increases survival in response to stress such as DNA damage by deacetylating a number of substrates including pro-apoptotic protein p53. The molecular mechanism by which DNA-damage activates SIRT1 is not known. By screening a kinase inhibitor library, we identified CK2 as a SIRT1 kinase. CK2 is a pleiotropic kinase with more than 300 substrates and well-known anti-apoptotic and pro-growth activities. We find that CK2 is recruited to SIRT1 after ionizing radiation (IR) and phosphorylates conserved residues Ser 154, 649, 651 and 683 in the N- and C-terminal domains of mouse SIRT1. Phosphorylation of SIRT1 increases its deacetylation rate but not if the four Ser residues are mutated. In addition, phosphorylation of SIRT1 increases its substrate-binding affinity. CK2-mediated phosphorylation increases the ability of SIRT1 to deacetylate p53 and protect cells from apoptosis after DNA damage. Based on these findings, we propose that CK2 protects against IR-induced apoptosis partly by phosphorylating and activating SIRT1. Thus, this work suggests that SIRT1 is a component of the expansive anti-apoptotic network controlled by CK2. Since expression of both CK2 and SIRT1 is upregulated with tumorigenesis and downregulated with senescence, the CK2-SIRT1 link sheds new light on how CK2 may regulate cancer development and aging.

## Introduction

Sirtuins [1], which are composed of seven members (SIRT1- SIRT7), are class III histone/protein deacetylases (HDAC). Unlike the other classes of HDACs, they require the coenzyme NAD+ (nicotinamide adenine dinucleotide) [2], [3]. Sirtuins are orthologs of yeast Sir2 (silencing information regulator) [2], [3], which mediates chromatin silencing [4], [5] and slows the aging rate by suppressing the production of extrachromosomal rDNA circles [6]. Within the Sirtuin family, SIRT1 [7] is most closely related to yeast Sir2. Calorie restriction, the only known intervention that extends life span in mammals, increases SIRT1 expression in some tissues [8], [9]. There is no evidence that SIRT1 regulates aging in mammals, but it does increase resistance to various forms of stress [8], [10], [11], [12], [13], [14]. One very well characterized pathway regulated by SIRT1 is DNA-damage induced apoptosis. SIRT1 deacetylates and decreases the transcriptional activity of pro-apoptotic p53 and increases cell survival after DNA damage [12], [15], [16], [17]. Consistent with the pro-survival function of SIRT1, p53 is hyperacetylated in mice lacking SIRT1, and thymocytes derived from these mice have increased sensitivity to IR [15]. However, the biochemical pathway that activates SIRT1 in response to DNA damage has not been discovered.

CK2, a tetrameric enzyme composed of two catalytic subunits (αα, αα' or α'α') and two regulatory β subunits, is a ubiquitously expressed and evolutionarily conserved serine/threonine protein kinase [18]. CK2α and CK2α' have approximately 90% identity in their catalytic domains and have similar enzymatic properties including substrate specificity. CK2 is a pleiotropic kinase that has more than 300 putative targets [19] and can be found in the nucleus, cytoplasm and specific structures and organelles such as the plasma membrane, Golgi, ribosomes and endoplasmic reticulum [20]. CK2 is essential for viability [21] and plays a critical role in tumor development [22]. Phosphorylation by CK2 prevents caspase-mediated cleavage of a number of proteins involved in the regulation of cell survival [23], and suppression of CK2 decreases cell proliferation and viability [24], [25]. Although CK2 has a high basal activity, it can be stimulated with growth factors [26], serum [27] and stresses such as DNA damage [28], [29]. CK2 function is also modulated by translocation to specific sites within the cell. For example, stresses such as ionizing radiation IR [25] and hypoxia [30] cause nuclear accumulation of CK2.

Here, we report that IR leads to CK2-SIRT1 interaction and CK2-mediated phosphorylation at four Ser residues. SIRT1 phosphorylation increases its substrate-binding affinity and its deacetylase activity. As a result, SIRT1 phosphorylation increases p53 deacetylation and survival after DNA-damage.

## Results and Discussion

### SIRT1 phosphorylation is CK2-dependent

**Figure 1:**
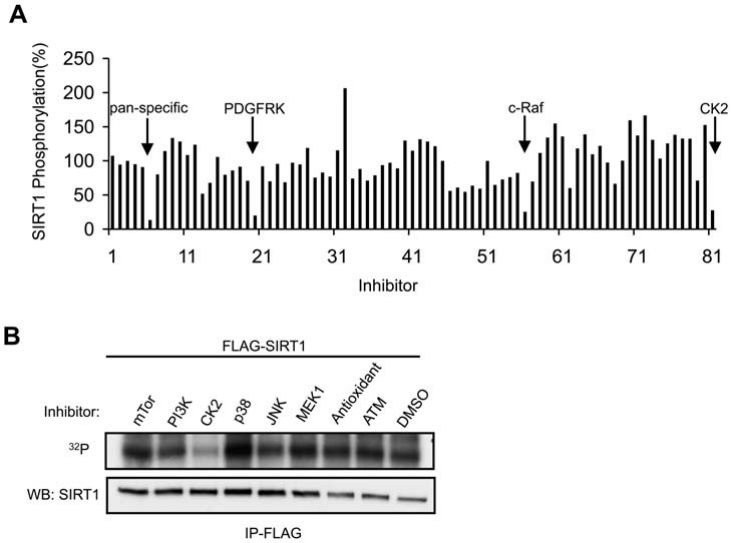
SIRT1 phosphorylation is CK2-dependent. (A) HEK 293T cells stably expressing Flag-tagged SIRT1 protein were incubated with ^32^P orthophosphoric acid and the indicated protein kinase inhibitor (10 µM) (see Table S1). Each bar represents the intensity of phosphorylated SIRT1 normalized to the total SIRT1 level after immunoprecipitation (IP) with M2 Flag antibody. The value of vehicle-treated sample was set to 100%. (B) In vivo ^32^P labeling of SIRT1 in the presence of inhibitors of stress-activated kinases that are indicated.

Phosphorylation is one of the most common mechanisms by which protein function is regulated. To identify a potential kinase that phosphoryates SIRT1, we screened a library of 81 kinase-inhibitors (10 µM) (Table S1) for their ability to inhibit ^32^P incorporation into SIRT1. We incubated HEK 293T cells stably expressing WT Flag-tagged mouse SIRT1 in 100 µCi/ml [^32^P]-orthophosphoric acid for one hr and isolated SIRT1 by immunoprecipitating with anti-FLAG antibody (Fig. 1A). From this screen, we identified four kinase-inhibitors that suppressed SIRT1 phosphorylation by more than 50%: Staurosporin (pan-specific), by 86.6%; Tyrphostin 9 (PDGF receptor kinase inhibitor), by 79.8%; GW 5074 (c-Raf inhibitor), by 74.5%; TBCA (the most potent CK2 inhibitor) [31], by 72.5%. Two other CK2 inhibitors used in this screen, DRB and Apigenin, did not significantly inhibit ^32^P incorporation because of weak inhibitory activity at the concentration that we used here. We also tested whether inhibitors of other growth or stress-related kinases that were not included in the screen affected ^32^P incorporation into SIRT1. As shown in Fig. 1B, only TBCA inhibited ^32^P incorporation into SIRT1.

The localization of PDGF receptor and c-Raf are limited to the plasma membrane and cytoplasm, respectively, whereas CK2 can be found in the nucleus, cytoplasm and specific structures and organelles such as the plasma membrane, Golgi, ribosomes and endoplasmic reticulum [20]. Although SIRT1 has been detected in both cytoplasm and nucleus in some cell types [32], [33], SIRT1 is a nuclear protein in most cell lines [34], [35], including the HEK 293T cell line that we used for this kinase inhibitor screen (data not shown). Based on common nuclear localization, we chose to investigate the possibility that CK2 is a SIRT1 kinase.

### Identification of CK2 phosphorylation sites in SIRT1

**Figure 2:**
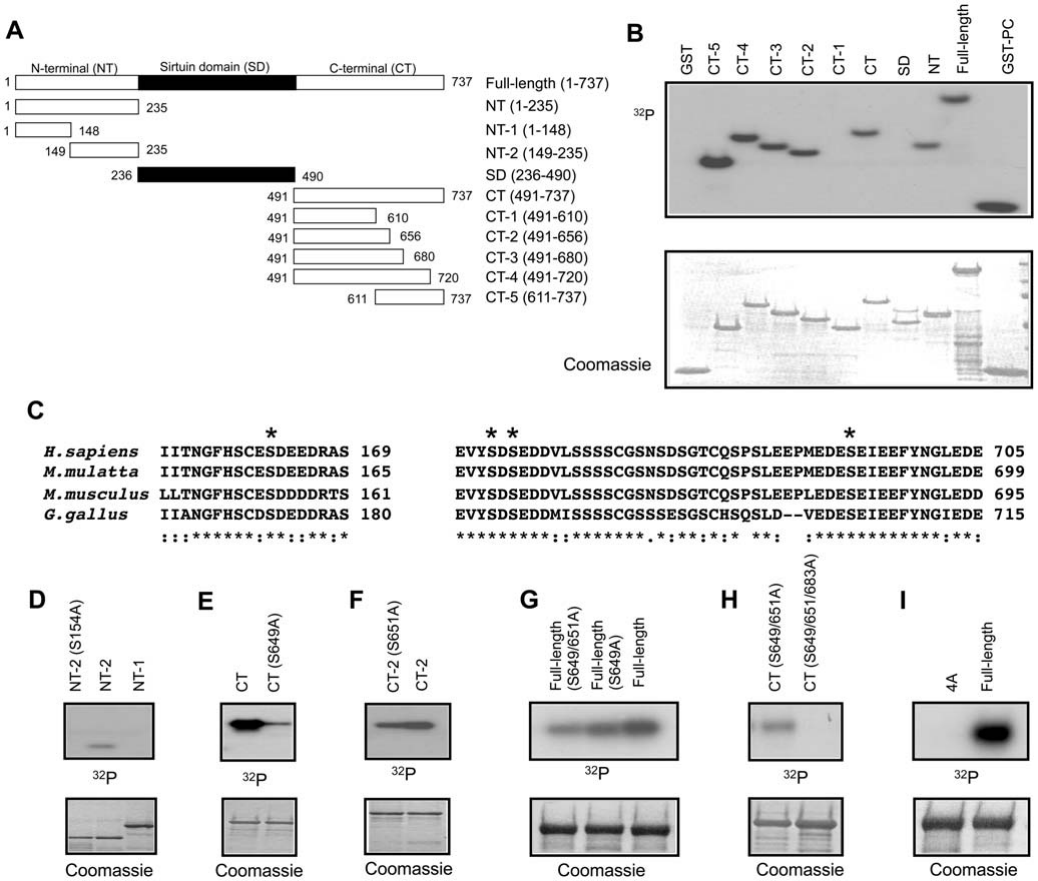
Identification of CK2 phosphorylation sites in mouse SIRT1. (A) Diagram of recombinant mouse SIRT1 fragments produced in E.coli. The a.a. number of the first and last a. a. for each fragment is as indicated. (B) SIRT1 fragments from (A) were incubated with CK2 α and ^32^P[γ-ATP], subjected to SDS-PAGE, Coomassie staining and autoradiography. GST-PC, which contains the optimized CK2 phosphorylation site, was used as a positive control. (C) Evolutionarily conserved CK2 consensus sites located in the fragments phosphorylated by CK2 are marked (*). (D–H) Indicated Ser (S) residues in SIRT1 fragments (see Fig. 2A ) were mutated to Ala (A) individually or in combination. Fragments containing the mutation were incubated with CK2 α and ^32^P[γ-ATP] and then subjected to SDS-PAGE, Coomassie staining and autoradiography. (I) CK2-dependent phosphorylation of full-length (FL) and S154/649/651/683A (4A) mutant SIRT1 *in vitro*. CK2 phosphorylation was performed and visualized as in (B).

To investigate whether CK2 phosphorylates SIRT1 directly, we produced fragments of mouse SIRT1 proteins in *E. coli* and performed *in vitro* kinase reactions using CK2 α(Fig. 2A). We found that CK2 phosphorylation sites were located in N-terminal (a.a. 1–235) and C-terminal (a.a. 611–737) domains but not in the Sirtuin (deacetylase) domain (Fig. 2B). The consensus sequence for CK2 phosphorylation is S/TXXD/E, where X can be any amino acid residue. There are four evolutionarily-conserved CK2 consensus sequences (Ser 154,649,651,683) within the CK2 phosphorylated regions of SIRT1 (*, Fig. 2C). To identify the CK2 phosphorylation sites, we mutated the four CK2 consensus sites to Ala either individually or in groups (Fig. 2D–I). Mutating Ser 154 to Ala (S154A) completely abolished CK2 phosphorylation of the N-terminal fragment indicating that Ser 154 is the only CK2 phosphorylation site in the N-terminal fragment (Fig. 2D). Mutating Ser 649 to Ala (S649A) in the C-terminal fragment significantly reduced SIRT1 phosphorylation but did not abolish it (Fig. 2E). Mutating Ser 651 to Ala (S651A) in fragment CT-2 consistently reduced SIRT1 phosphorylation, albeit slightly (Fig. 2F). Mutating Ser 649 and 651 to Ala decreased phosphorylation of full-length SIRT1 more than mutating Ser 649 alone, indicating that both Ser 649 and 651 are phosphorylated by CK2 (Fig. 2G). Mutating Ser 649, 651 and 683 to Ala in the C-terminal fragment completely abolished phosphorylation of the C-terminal fragment but mutating Ser 649 and 651 did not, indicating that Ser 683 is also phosphorylated by CK2 (Fig. 2H). Mutating Ser 154, 649, 651 and 683 (4A) completely abolished CK2 phosphorylation of full-length SIRT1 (Fig. 2I), indicating that CK2 phosphorylates all four conserved Ser residues *in vitro*.

### CK2-mediated phosphorylation of SIRT1 *in vivo*

**Figure 3:**
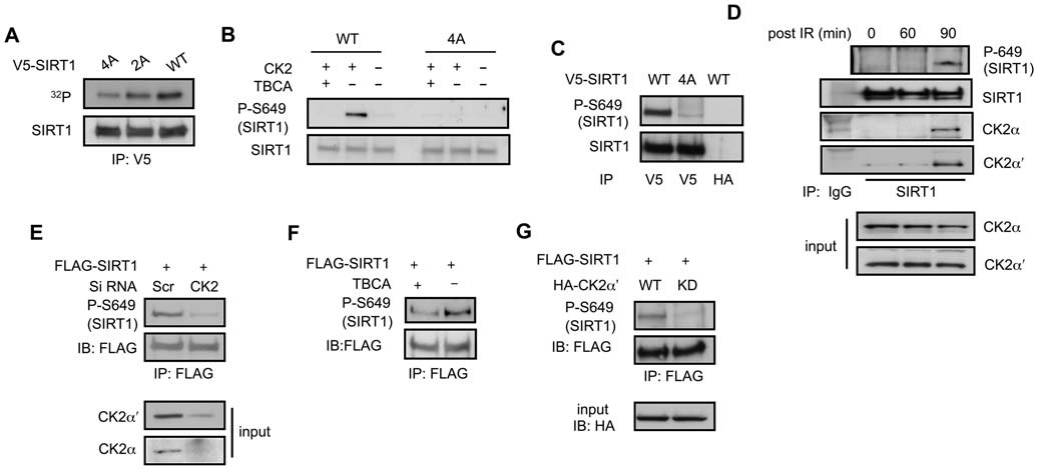
CK2 phosphorylates SIRT1 after ionizing radiation. (A) V5-tagged WT SIRT1, 2A (S649/651A) SIRT1 and 4A (S154/649/651/683A) SIRT1 expression plasmids were transfected into HeLa cells and labeled with ^32^P orthophosphoric acid for 2 hrs. After labeling, V5-SIRT1 was immunoprecipitated. ^32^P-labeled SIRT1 was detected by autoradiography and the total level of SIRT1, by immunoblotting (IB) with SIRT1 antibody. (B) Recombinant full-length WT and 4A SIRT1 were phosphorylated by CK2 with DMSO (−) or with TBCA (20 µM). Immunoblotting was performed with S649-specific phospho-antibody (P-S649). Total SIRT1 levels are shown below. (C) Transiently expressed V5-WT or 4A SIRT1 was immunoprecipitated with V5 antibody and immunoblotted with P-S649 antibody. Immunoprecipitation with HA antibody was used as a control. (D) HeLa cells were harvested 60 and 90 min after IR (20 Gy) and endogenous SIRT1 was immnoprecipitated with either SIRT1 antibody or a control antibody, IgG. Immunoblotting was performed with P-S649 antibody to visualize SIRT1 phosphorylation and with SIRT1 antibody to visualize total SIRT1 levels. SIRT1-CK2 interaction was visualized by immunoblotting with antibody specific for CK2 α or CK2 α'. (E) Flag-tagged WT SIRT1 transiently expressed in HeLa cells with siRNA for either a scrambled sequence (Scr) or both catalytic subunits of CK2. Two hr after IR (20 Gy), SIRT1 was immunoprecipitated and then immunoblotted with P-S649 to visualize CK2 phosphorylation. (F) Lysates from HeLa cells co-expressing Flag-tagged WT SIRT1 treated with DMSO (−) or with 20 µM TBCA (+) starting 1 hr before IR (20 Gy). Two hr after IR, SIRT1 was immunoprecipitated with Flag antibody and then immunoblotted with P-S649 antibody. (G) Lysates from HeLa cells co-expressing Flag-tagged WT SIRT1 and either WT or KD CK2 were harvested 2 hr after IR. SIRT1 was immunoprecipitated with a Flag antibody and then immunoblotted with P-S649 antibody.

To confirm that the four Ser residues were phosphorylated *in vivo*, we performed the [^32^P]-orthophosphoric acid incorporation assay after transiently expressing wild-type (WT) and 2A (S649/651A) and 4A (S154/649/651/683A) mutant SIRT1 in HEK 293T cells (Fig. 3A). Compared with WT SIRT1, the 2A and 4A mutants showed progressively decreasing phosphorylation intensity. Even in 4A mutant, there was some residual phosphorylation, indicating that other kinase(s) also phosphorylate SIRT1 *in vivo*. To directly visualize phosphorylation at these four Ser residues, we attempted to make phospho-specific antibodies recognizing each of the four phosphorylated Ser residues. However, due to the acidic nature of some of these sites, we were able to make a phospho-specific antibody only for Ser 649 (Ser 659 in human SIRT1). To test the specificity of the phospho-S649 (P-S649) antibody, we performed CK2 kinase reactions using either WT or 4A SIRT1 as substrates with or without TBCA in the reaction mixture. As shown in Fig. 3B, P-S649 antibody recognized SIRT1 phosphorylated by CK2 *in vitro* but not 4A SIRT1. P-S649 antibody also recognized WT but not 4A SIRT1 in transiently transfected cells (Fig. 3C). Ser 649 phosphorylation of endogenous SIRT1 was poorly visible in untreated cells but was induced 90 min after IR (Fig. 3D). The interaction between SIRT1 and the two catalytic subunits of CK2 also increased significantly 90 min after IR (Fig. 3D). To demonstrate that SIRT1 phosphorylation is mediated by CK2, we transiently expressed SIRT1 along with siRNA for either a scrambled sequence or for both catalytic subunits of CK2 (α and α'). Two hours before they were harvested, cells were exposed to IR to induce Ser 649 phosphorylation. Knock-down of CK2 dramatically reduced Ser 649 phosphorylation, indicating that CK2 phosphorylates Ser 649 *in vivo* (Fig. 3E). In agreement with this, treatment of cells with CK2-inhibitor TBCA (Fig. 3F) or coexpression of kinase-dead (KD) CK2 α' (Fig. 3G) significantly inhibited Ser 649 phosphorylation. Our inability to detect Ser 649 phosphorylation in endogenous SIRT1 without IR (Fig. 3D) was unexpected because CK2 has high basal activity and also because we did not use IR when we discovered through our kinase inhibitor screen (Fig. 1) that TBCA inhibited SIRT1 phosphorylation. We believe that this apparent contradiction is due to the low sensitivity of P-S649 antibody. Ser 649 phosphorylation is visible in transiently expressed SIRT1 because there is more SIRT1 protein in the cell and the vector DNA triggers the DNA-break signal in the way IR does (data not shown).

**Figure 4:**
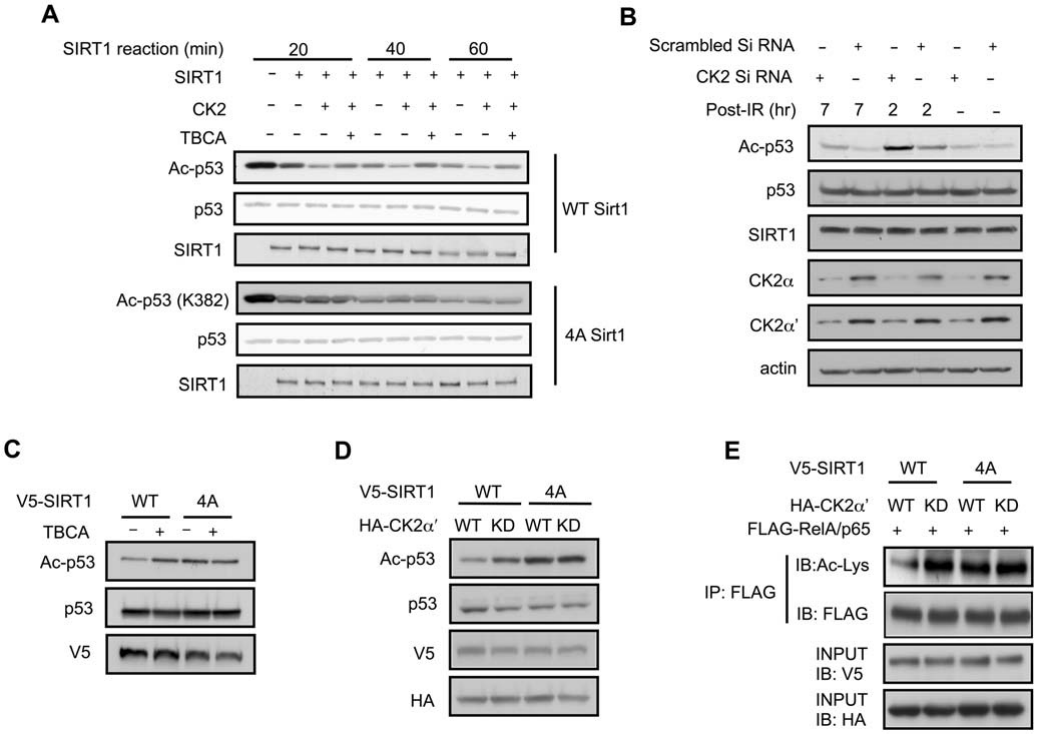
CK2 phosphorylation stimulates SIRT1 activity. (A) Deacetylase activity of recombinant WT or 4A SIRT1 that was phosphorylated with CK2 in the presence or absence of TBCA (20 µM). Deacetylase activity of SIRT1 was visualized by immunoblotting with acetylated p53 (K382) antibody. The total level of p53 is visualized by Ponceau staining the total level of SIRT1 is visualized by immunoblotting with SIRT1 antibody. (B) The deacetylase activity of the endogenous SIRT1 in the presence of siRNA for either a scrambled sequence or for both catalytic subunits of CK2. P53 K382 acetylation was visualized 2 and 7 hr after IR (20Gy) in U2OS cells. Total levels of p53, SIRT1, CK2α, CK2α' and actin are shown below. (C) H1299 cells co-expressing V5-tagged WT or 4A SIRT1 and p53 in the presence or absence of TBCA were harvested 6 hr after IR (20 Gy). Acetylation of p53 was visualized by immunoblotting with acetylated p53 (K382) antibody. (D) V5-tagged WT or 4A SIRT1 was transiently co-expressed with p53 and either HA-tagged WT or KD CK2α' in H1299 cells. Cells were harvested 6 hr after IR and deacetylation of p53 was visualized by immunoblotting with acetylated p53 (K382) antibody. (E) V5-tagged WT or 4A SIRT1 was transiently co-expressed with FLAG-RelA/p65 (a component of NF-κB) and either HA-tagged WT or KD CK2α' in H1299 cells. FLAG-RelA/p65 was immunoprecipitated with FLAG antibody and immunoblotted with either FLAG antibody to visualize the total level of FLAG-RelA/p65 or Ac-Lys antibody to visualize the acetylation level.

To determine how phosphorylation by CK2 affects SIRT1, we measured the deacetylase activity of SIRT1 after CK2 phosphorylation *in vitro*. Deacetylation of Lys 382 in p53, which is known to be mediated by SIRT1 [16], was used as the indicator for SIRT1 activity. We monitored p53 deacetylation after 20, 40 or 60 min reaction time (Fig. 4A). For all three reaction times, deacetylation of p53 by WT SIRT1 was increased after CK2 phosphorylation, but not if TBCA was present during the phosphorylation reaction. However, deacetylation of p53 by 4A SIRT1 was not affected by CK2. It should be noted that in the absence of CK2 phosphorylation, the deacetylase activity of 4A SIRT1 is comparable to that of WT SIRT1, indicating that the four Ser residues are not essential for SIRT1 activity *in vitro*.

### Phosphorylation by CK2 activates SIRT1

In response to DNA damage, Lys 382 in p53 is acetylated by histone acetyltransferase p300 [36], [37]. Acetylation of p53 increases its affinity to the target DNA sequence and thus its transcriptional activity. We investigated the effect of CK2 on SIRT1 activity in U2OS cells using p53 (K382) acetylation as the endogenous marker for the deacetylase activity of SIRT1. The acetylation level of K382 at any given moment is determined by the rate of acetylation and deacetylation at that moment. To determine the time course of CK2-mediated activation of SIRT1, we visualized acetylation of K382 at different time points after IR in cells transfected with siRNA for either a scrambled sequence or for both catalytic subunits of CK2 (Fig. 4B). Consistent with the observation that acetylation of K382 by p300 occurs 2 hr after IR [37], K382 acetylation peaked 2 hr after IR. However, K382 acetylation at 2 hr was higher when CK2 was knocked-down and did not return to baseline even at 7 hr, indicating that CK2 increases the deacetylase activity of SIRT1. Although CK2 enhanced SIRT1 activity at 2 hr point, the acetylation level of K382 at this time is still higher than before IR, suggesting that p300 activity exceeds SIRT1 activity at 2 hr point. Although this experiment suggests that CK2-activated SIRT1 deacetylated p53, we cannot rule out the possibility that activated SIRT1 decreased p53 acetylation by an indirect mechanism, such as improved DNA repair leading to reduced p300-mediated acetylation of p53. However, as of now, there is no evidence that SIRT1 improves DNA repair.

In order to confirm that CK2 phosphorylation increases SIRT1 activity, we transiently expressed p53 along with WT SIRT1 or 4A SIRT1 in H1299 cells and monitored p53 acetylation. The deacetylase activity of WT SIRT1 was inhibited in the presence of TBCA but the deacetylase activity of 4A SIRT1, which was lower than that of WT SIRT1, was insensitive to TBCA (Fig. 4C). Since there is a possibility that the effect of TBCA on SIRT1 activity was due to an off-target hit of TBCA, we overexpressed kinase-dead (KD) CK2 α' to inhibit the CK2 activity in a dominant-negative fashion (Fig. 4D). The deacetylase activity of WT SIRT1 as measured by p53 deacetylation was suppressed in the presence of KD CK2 α', but the deacetylase activity of 4A SIRT1 was not affected by KD CK2 α'. To determine if CK2 regulates deacetylation of other SIRT1 substrates, we repeated the experiment using RelA/p65 subunit of NF-κB, another known SIRT1 substrate (Fig. 4E) [38]. The deacetylase activity of WT SIRT1, as measured by RelA/p65 deacetylation, was suppressed in the presence of KD CK2 α', but the deacetylase activity of 4A SIRT1 was not affected by KD CK2 α'. We also tried to knock-down CK2 using siRNA specific for the catalytic subunits of CK2 in cells transiently expressing either WT or 4A SIRT1. However, we noticed that the combination of CK2 siRNA and 4A SIRT1 expression caused cell death, and therefore did not continue this experiment. Thus, CK2-dependent phosphorylation of SIRT1 stimulates the deacetylase activity of SIRT1 against at least two known substrates, p53 and NF-κB.

### Phosphorylation by CK2 increases SIRT1 substrate-binding

**Figure 5:**
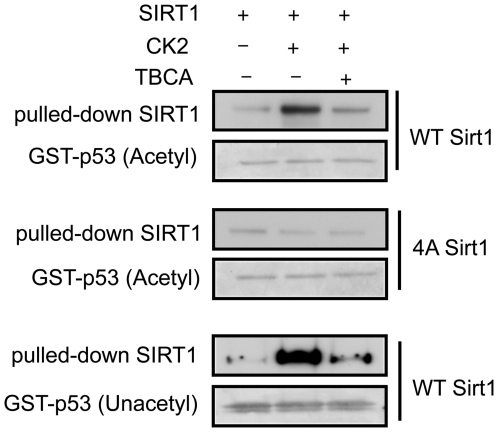
Phoshorylation by CK2 increases SIRT1-p53 interaction. GST-pull down experiment was performed with GST-p53 (acetylated) using WT (top) and 4A SIRT1 (center) phosphorylated with CK2 with or without TBCA. The same experiment using unacetylated GST-p53 and WT SIRT1 is shown at the bottom.

Since the CK2 phosphorylation sites are distant from the Sirtuin domain of SIRT1, it is curious that deacetylation of its substrate is increased by CK2 phosphorylation. One possibility is that SIRT1 phosphorylation changes its substrate binding affinity. To investigate whether phosphorylation by CK2 affects SIRT1-substrate interaction, we performed a GST-pull down assay using acetylated GST-p53 fusion protein and WT or 4A mutant SIRT1 that has been preincubated with CK2. As shown in Fig. 5, preincubation of WT SIRT1 with CK2 increased WT SIRT1-p53 interaction in a TBCA-sensitive manner. In contrast, preincubation of 4A SIRT1 with CK2 did not affect SIRT1-p53 interaction. To determine if acetylation of the substrate is required for the increased SIRT1-p53 interaction, we also performed the GST-pull down assay using unacetylated GST-p53. CK2-mediated phosphorylation increased SIRT1 interaction with unacetylated GST-p53 (Fig. 5, bottom), indicating that the acetyl-group on the substrate is not a feature that is required for the enhanced affinity. Taken together, these findings indicate that one mechanism by which CK2-mediated phosphorylation increases SIRT1 deacetylase activity is increasing interaction with its substrate. We cannot rule out the possibility that CK2-mediated phosphorylation also increases SIRT1 activity by other mechanisms.

### Anti-apoptotic function of SIRT1 is enhanced by CK2-mediated phosphorylation

**Figure 6:**
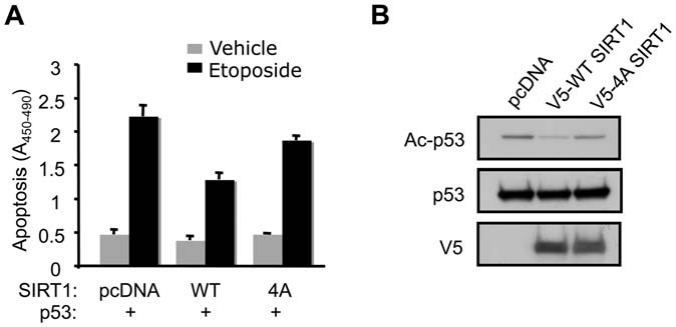
CK2 phosphorylation confers increased SIRT1-mediated survival after genotoxic treatment. (A) H1299 cells transiently expressing p53 and either WT or 4A SIRT1 were treated with the genotoxin etoposide (20 µM) for 24 hr. Apoptosis was quantified by cell death detection ELISA^PLUS^. Data are represented as mean±SEM. **, p<0.01 between WT SIRT1 and pcDNA (n = 3). #, p<0.05 between 4A SIRT1 and pcDNA (n = 3). (B) Deacetylation of p53 in etoposide-trated cells was visualized in cells expressing p53 and either WT or 4A SIRT1 by immunoblotting with acetylated p53 (K382) antibody.

SIRT1 protects cells from apoptosis induced by DNA damage [11], [12], [14], [16]. The most commonly studied role of SIRT1 in stress resistance is p53 deacetylation and suppression of p53-induced apoptosis. To measure the effect of CK2-mediated phosphorylation of SIRT1 on p53 mediated apoptosis, H1299 cells were co-transfected with p53 and either WT or 4A SIRT1 and treated with the genotoxin etoposide for 24 hr. Quantification of apoptosis indicated that WT SIRT1 suppressed etoposide-induced apoptosis by 42% and 4A SIRT1, by 16%, compared to vector (pcDNA)-alone control (Fig. 6A). Thus, the CK2 phosphorylation sites are important for the anti-apoptotic effects of SIRT1. Since the 4A mutation does not significantly affect the intrinsic SIRT1 activity (see Fig. 4A), the low magnitude of the anti-apoptotic effect of 4A SIRT1 is most likely due to the baseline protection from endogenous SIRT1 in all the samples. Deacetylation of p53 was significantly stronger with WT SIRT1 than with 4A SIRT1 (Fig. 6B) in these cells. Collectively, these results suggest that the anti-apoptotic function of SIRT1 is enhanced by CK2-mediated phosphorylation.

Despite the prominent role of SIRT1 in DNA damage-response, the signaling pathway that activates SIRT1 after DNA-damage was unknown. Our results indicate that CK2 activates SIRT1 by phosphorylating four Ser residues in N- and C-terminal domains. Although very little is known about the function of the N- and C-terminal domains of SIRT1, our finding that CK2-mediated phosphorylation increases SIRT1-p53 interaction suggests that the N- and C-terminal domains regulate substrate recruitment. A full understanding of how these terminal domains and their phosphorylation regulate SIRT1 function is hampered by the lack of 3D structure information on the full-length SIRT1 or on the terminal domains. It remains to be determined whether CK2-mediated phosphorylation affects SIRT1 activity by simply increasing substrate recruitment or whether other mechanisms are also involved.

To our knowledge, this work represents the first identification of a signal pathway that activates the catalytic function of SIRT1 in response to DNA damage. While this work was in progress, Zschoernig et. al. [39] reported that CK2 phosphorylates Ser 649/651 but did not determine whether phosphorylation affected SIRT1 in any way or under what conditions CK2 phosphorylates SIRT1. Also, Sasaki et. al. [40] reported that SIRT1 is activated by phosphorylation at multiple sites and two of these sites (Thr 530 and Ser 540 in human SIRT1) are phosphorylated by cyclinB/Cdk1, and mutation of these residues disturbed cell cycle progression. However, phosphorylation by cyclinB/Cdk1 *in vitro* did not affect the catalytic activity of SIRT1. Thus, it is not clear how phosphorylation by cyclinB/Cdk1 activates SIRT1 activity. Interestingly, SIRT1 activity is activated by sumoylation of Lys 734 [13]; and DNA-damage causes SENP1-mediated desumoylation and inhibition of SIRT1 activity. SIRT1 activity is also regulated by proteins that bind SIRT1 directly. AROS, which binds to the N-terminal domain of SIRT1, activates SIRT1 [41]. It is not known if AROS-SIRT1 interaction is regulated by DNA damage or other forms of stress. In contrast, DBC1, which binds to the Sirtuin domain of SIRT1, inhibits SIRT1 activity in response to DNA-damage [11], [14]. Thus, these previous reports and our findings indicate that DNA-damage has both inhibitory and stimulatory effects on SIRT1 activity.

Previous studies have shown that CK2 can phosphorylate Ser 392 [42] of p53 and enhance its activity by increasing tetramerization and DNA binding [43]. Therefore, CK2 may regulate p53 activity directly and indirectly through SIRT1 as we have demonstrated here. However, Ser 392 is phosphorylated after UV irradiation but not after IR or etoposide [28], [44], suggesting that CK2 does not phosphorylate p53 in response to double-stranded breaks. Since we utilized IR and etoposide, not UV, in this work, it is unlikely that direct p53 phosphorylation played a significant role in CK2-dependent regulation of p53 that we describe here.

Our findings show that SIRT1 is a component of an expansive anti-apoptotic network that is controlled by CK2. Since expression of both CK2 and SIRT1 is upregulated with tumorigenesis [22], [45], [46] and downregulated with senescence [47], [48], the CK2-SIRT1 pathway is likely to play an important role in regulating cellular survival and death associated with cancer and aging. Although SIRT1 has anti-apoptotic functions, it may also have anti-tumorigenesis functions as well. SIRT1 is important for genetic stability [49], and when it is overexpressed in colonic epithelial cells, it deacetylates β-catenin and suppresses colon cancer cell growth [50]. Thus, SIRT1 appears to mediate a delicate balance between its anti-growth and pro-growth properties.

## Materials and Methods

### Protein kinase inhibitor library screening

The kinase inhibitors were derived from the Protein kinase inhibitor library (BIOMOL international) and other commercial sources. HEK 293T cells stably expressing WT Flag-tagged mouse SIRT1 were seeded onto 6 well plates at 5×10^6^ cells per well. One day after plating, cells were pre-incubated with phosphate free DMEM media (Gibco) containing 10 µM protein kinase inhibitor for 1 hr. After pre-incubation, 100 µCi/ml [^32^P]-orthophosphoric acid was added into each well directly and incubated for another 2 hr. Labeled Flag-tagged SIRT1 proteins were immunoprecipitated with Flag M2-Agarose (Sigma), separated by 4–12 % SDS-PAGE and transferred to nitrocellulose. The amounts of [^32^P]-labeled and loaded SIRT1 proteins were detected by autoradiography and immunoblotting with SIRT1 antibody, respectively.

### In vitro kinase assay

To identify CK2 phosphorylation sites, recombinant full-length or truncated His-tagged or GST-tagged SIRT1 proteins were incubated in kinase buffer (20 mM HEPES at pH 7.0, 10 mM MgCl_2_, 1 mM DTT) containing 100 µM cold ATP, 1 µCi (3000 mCi/mmole) ^32^P[γ-ATP] and 20 ng CK2α enzyme (Millipore) for 20 min at 30°C. Where indicated, TBCA (20 µM) dissolved in DMSO was added to the reaction mixture.

### In vitro deacetylase assay

For in vitro deacetylase assays, 2 µg purified His-tagged WT and 4A mutant full-length SIRT1 proteins were incubated with CK2α enzyme with or without 20 µM TBCA in a kinase buffer described above except radioactive ATP was not included. After the kinase reaction, the samples were loaded onto CENTRISEP spin columns (Princeton separations) pre-equilibrated with 1×PBS to remove the excess ATP and free additives in CK2 α enzyme solution. 1/10 volume of above phosphorylated or mock-phosphorylated SIRT1 proteins were added to a deacetylase buffer (50 mM HEPES at pH 7.0, 1 mM DTT, 150 mM NaCl, protease inhibitor cocktail, and phosphatase inhibitor cocktail (Roche)) containing 0.2 µg acetylated GST-p53, and 0.5 mM NAD^+^ in a 20 µl final reaction volume. The reactions were performed at 37°C for the indicated durations and stopped by adding SDS sample buffer. Heated samples were subjected to 4–12% SDS PAGE, transferred to nitrocellulose, and immunoblotted with Ac-p53 (K382) antibody (Cell Signaling Technologies). The loading amounts of SIRT1 and GST-p53 proteins were visualized with Coomassie stain and Ponceau S stain.

### Acetylation of GST-p53 protein

Glutathione S-transferase (GST) fused to human p53 fragment (amino acids 373–385) was expressed in *E. coli* and purified on glutathione Sepharose beads according to the vendor's manual. Purified GST-p53 was acetylated by His-tagged active core p300/CBP associated factor (PCAF, amino acids 352–832) in HAT buffer (Millipore) containing 5 mM acetyl CoA. As shown in Fig. S1, K382 was strongly acetylated by this method. After incubating for 3 hrs, the reaction mixture was incubated with glutathione Sepharose and acetylated GST-p53 proteins were purified and dialyzed against 20 mM HEPES buffer (pH 7.0) containing 2 % glycerol, 1 mM DTT, and 50 mM NaCl.

### GST-Pull down assay

To measure the interaction between mouse SIRT1 and p53 peptide, 0.2 µg GST-p53 and 0.2 µg mouse SIRT1 (preincubated with CK2 with or without TBCA) was incubated with glutathione-agarose beads (GE) in lysis buffer containing 150 mM NaCl

for 2 hr at room temperature. The beads were washed once with lysis buffer and twice with RIPA buffer containing 300 mM NaCl. Proteins retained on the beads were eluted with 2X SDS sample buffer and subjected to SDS-PAGE. Bound SIRT1 was visualized by immunoblotting and GST-p53 was visualized by Ponceau staining.

### Proteins, Cell lines and Expression constructs

Wild-type and alanine mutant His-tagged mouse SIRT1 were constructed in pET-15b prokaryotic expression vector using NdeΙ and XhoΙ restriction sites, expressed in E.coli, and purified on Ni^2+^-NTA beads (Qiagen) following the manufacturer's protocol. GST fusion constructs of full-length and truncated mouse SIRT1 were generated by PCR amplification and subcloned into pGEX 4T-3 vector using SalΙ and XhoΙ sites. All GST-fusion constructs were transformed into BL21 competent cells and the recombinant proteins were purified and dialyzed as described above. To produce GST-PC, optimized CK2 substrate (Fig. 2B), nucleotides corresponding to RRRDDDSDDD were ligated into pGEX 4T-3 vector using Bam H1 and XhoΙ sites. To produce the mammalian SIRT1 expression vectors, WT and mutant mouse SIRT1 coding sequences were subcloned into pcDNA6.0 (Invitrogen) and FLAG-CMV expression vector (Sigma) using KpnΙ and XhoΙ sites. Wild-type and kinase-dead dominant negative CK2 α' expression vectors were obtained from Dr. Litchfield (the University of Ontario, Canada). H1299 non-small cell lung cancer cells, Hela cervical cancer cells, HEK 293T cells, Hepa 1–6 mouse hepatoma cell, and U2OS human osteosarcoma cells were maintained in DMEM medium supplemented with 10% FBS. The expression vector for FLAG-RelA/65 (1–313) was previously published [38].

### Apoptosis assay

To measure p53-dependent apoptosis, we co-transfected H1299 cells with expression vectors for p53 and pcDNA (empty), WT or 4A mutant SIRT1 and K^K^ plasmid. Transfected cells were selected using autoMax system (Miltenyi Biotec Inc.) 36 hr after transfection. After a further 24 hr, the cells were treated with etoposide (20 µM) for 24 hr and apoptosis was measured by the cell death detection ELISA Plus (Roche) according to the instruction manual.

### Immunoblotting and immunoprecipitation assays

Whole cell lysates were prepared by adding a lysis buffer containing phosphate buffered saline supplemented with 1 % Triton X-100, protease and phosphatase inhibitor cocktails (Roche). Extracts were separated by 4–12 % SDS-PAGE gels, transferred to nitrocellose mambrane and probed with primary antibodies as indicated. To immunoprecipitate, cells treated as indicated were extracted with lysis buffer and incubated with recommended amount of primary antibodies by manufacturer for overnight at 4°C and then further incubated with protein A/G agarose beads (Pierce) for 2 hrs. These beads were loaded onto Micro Bio-Spin chromatography columns (Bio-Rad), washed four times with lysis buffer, which was then removed by centrifugation. Immunoprecipitated proteins were denatured with SDS-sample buffer and released from the column by centrifugation. Antibodies for SIRT1 (Upstate Biotechnology), Acetyl-Lys (Cell Signaling Technology), CK2α and α' (Santa Cruz Biotechnology) were purchased from commercial sources.

### P-S649 antibody generation

The phosphospecific antibody was manufactured by 21st Century Biochemicals (Marlborough, MA). Briefly, a peptide corresponding to the sequence C-Ahx-IFHGAEVY[pS]D-amide was manufactured by Fmoc chemistry, HPLC purified to>90%, and the mass and sequence verified by nanospray MS and CID MS/MS, respectively. This peptide was conjugated to a carrier protein and was used to immunize two New Zealand rabbits. After multiple boosts and bleeds, serum that detected the target via western blots was pooled, immunodepleted by passing the serum over cross-linked agarose conjugated with unmodified peptide, and subsequently affinity purified using phosphopeptide-cross-linked agarose.

### Si RNA transfection

SMART pool siRNA (Dharmacon, Inc.) for both CK2 α and α' were transfected into HEK293 T cells using Lipofectamine 2000. The siRNA sequences for CK2 α is: GCAUUUAGGUGGAGACUUC, GGAAGUGUGUCUUAGUUAC, GCUGGUCGCUUACAUCACU and AACAUUGUCUGUACAGGUU. The siRNA sequences for CK2 α' is: GAGUUUGGGCUGUAUGUUA, GGGACAACAUUCACGGAAA, GAUAGAUCACCAACAGAAA and UUAAGCAACUCUACCAGAU.

## Supporting Information

Table S1 A list of kinase inhibitors used to screen for Sirt1 kinases.
(0.07 MB PDF)

Figure S1 GST-p53 acetylated by PCAF was immunoblotted with acetyl-K382 antibody.
(0.10 MB PDF)
