# Supplementary material for: CK2 Is the Regulator of SIRT1 Substrate-Binding Affinity, Deacetylase Activity and Cellular Response to DNA-Damage
Source: PLoS One. 2009 Aug 14;4(8):e6611. doi: 10.1371/journal.pone.0006611 (PMC2721681; doi:10.1371/journal.pone.0006611)
Supplement: Table S1 — A list of kinase inhibitors used to screen for Sirt1 kinases. (0.07 MB PDF) [file pone.0006611.s001.pdf]

No	compound name	Target	No	compound name	Target
1	PD-98059	MEK	42	ML-7	MLCK
2	U-0126	MEK	43	ML-9	MLCK
3	SB-203580	p38 MAPK	44	2-Aminopurine	p58 PITSLRE beta1
4	H-7	PKA, PKG, MLCK, and PKC.	45	N9-Isopropyl-olomoucine	CDK
5	H-9	PKA, PKG, MLCK, and PKC.	46	Olomoucine	CDK
6	Staurosporine	Pan-specific	47	iso-Olomoucine	Negative control for olomoucine.
7	AG-494	EGFRK, PDGFRK	48	Roscovitine	CDK
8	AG-825	HER1-2	49	5-Iodotubercidin	ERK2, adenosine kinase, CK1, CK2,
9	Lavendustin A	EGFRK	50	LFM-A13	BTK
10	RG-14620	EGFRK	51	SB-202190	p38 MAPK
11	Tyrphostin 23	EGFRK	52	PP2	Src family
12	Tyrphostin 25	EGFRK	53	ZM 336372	cRAF
13	Tyrphostin 46	EGFRK, PDGFRK	54	SU 4312	Flk1
14	Tyrphostin 47	EGFRK	55	AG-1296	PDGFRK
15	Tyrphostin 51	EGFRK	56	GW 5074	cRAF
16	Tyrphostin 1	Negative control for tyrosine kinase inhibitors.	57	Palmitoyl-DL-carnitine Cl	PKC
17	Tyrphostin AG 1288	Tyrosine kinases	58	Rottlerin	PKC delta
18	Tyrphostin AG 1478	EGFRK	59	Genistein	Tyrosine Kinases
19	Tyrphostin AG 1295	Tyrosine kinases	60	Daidzein	Negative control for Genistein.
20	Tyrphostin 9	PDGFRK	61	Erbstatin analog	EGFRK
21	HNMPA	IRK	62	Quercetin dihydrate	PI 3-K
22	PKC-412	PKC inhibitor	63	SU1498	Flk1
23	Piceatannol	Syk	64	ZM 449829	JAK-3
24	PP1	Src family	65	BAY 11-7082	IKK pathway
25	AG-490	JAK-2	66	DRB	CK2
26	AG-126	IRAK	67	HBDDE	PKC alpha, PKC gamma
27	AG-370	PDGFRK	68	SP 600125	JNK
28	AG-879	NGFRK	69	Indirubin	GSK-3beta, CDK5
29	LY 294002	PI 3-K	70	Indirubin-3'-monoxime	GSK-3beta
30	Wortmannin	PI 3-K	71	Y-27632	ROCK
31	GF 109203X	PKC	72	Kenpaullone	GSK-3beta
32	Hypericin	PKC	73	Terreic acid	BTK
33	Ro 31-8220	PKC	74	Triciribine	Akt signaling pathway
34	Sphingosine	PKC	75	BML-257	Akt
35	H-89	PKA	76	SC-514	IKK2
36	H-8	PKA, PKG	77	BML-259	Cdk5/p25
37	HA-1004	PKA, PKG	78	Apigenin	CK2
38	HA-1077	PKA, PKG	79	BML-265	EGFRK
39	HDBA	EGFRK, CaMK II	80	Rapamycin	mTOR
40	KN-62	CaMK II	81	TBCA	CK2
41	KN-93	CaMK II			

Table S1 List of kinase inhibitors used for SIRT1-kinase screen. The names of the inhibitors and the kinases targeted by the inhibitors are shown.
