# Supplementary figures and images for: CK2 Is the Regulator of SIRT1 Substrate-Binding Affinity, Deacetylase Activity and Cellular Response to DNA-Damage
Source: PLoS One. 2009 Aug 14;4(8):e6611. doi: 10.1371/journal.pone.0006611 (PMC2721681; doi:10.1371/journal.pone.0006611)

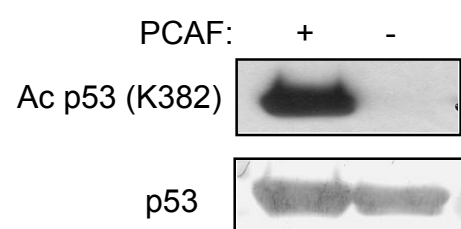

**Figure. S1. Acetylation of p53 K382.** GST-p53 acetylated by PCAF was immunoblotted with acetyl-K382 antibody.

Supplement: Figure S1 — GST-p53 acetylated by PCAF was immunoblotted with acetyl-K382 antibody. (0.10 MB PDF) [file pone.0006611.s002.pdf]
